# Unveiling the mechanisms driving the rapid growth of *Malania oleifera* seedlings, a high-value root hemiparasitic plant

**DOI:** 10.3389/fpls.2025.1589651

**Published:** 2025-07-17

**Authors:** Si-Hai Wang, Chuan-Guang Zhang, Wei Yang, Jian Chen, Ming Shi

**Affiliations:** ^1^ Yunnan Provincial Key Laboratory of Forest Plant Cultivation and Utilization, Yunnan Academy of Forestry and Grassland, Kunming, China; ^2^ Key Laboratory of the State Forestry Administration on Conservation of Rare, Endangered and Endemic Forest Plants, Kunming, China; ^3^ College of Forestry, Southwest Forestry University, Kunming, China

**Keywords:** haustorium, host plant, biomass, phytohormone, transcriptome analysis, soil conditions

## Abstract

Certain root hemi-parasitic tree species hold significant economic value, yet they are challenging to cultivate artificially. Therefore, understanding how soil conditions and host plants influence the growth of these species is crucial. The endemic tree species *Malania oleifera*, native to the karst landscapes of southwest China, is highly valued for its seed oil, rich in nervonic acid. As a root hemiparasite, *M. oleifera* presents challenges for artificial cultivation, making it crucial to improve seedling survival and develop effective propagation methods for this and similar species. We used nutrient-rich and nutrient-deficient growth substrates, combined with four planting configurations involving host and non-host plants, to monitor the growth of *M. oleifera* seedlings. We then analyzed the transcriptomic differences between non-parasitic and parasitic plants that exhibited significant growth disparities. Vigorous host plants significantly enhance the growth of *M. oleifera* seedlings, while soil conditions exert a weaker influence. The host primarily promotes aboveground *M. oleifera* growth, with only limited impact on root development, resulting in an imbalance between the two. Endogenous hormone levels in the haustoria connected to the host exhibit substantial changes, with notable upregulation of genes related to hormone metabolism, stress responses, and antibiotic biosynthesis. Furthermore, the roots of host-associated *M. oleifera* seedlings show heightened responses to both biotic and abiotic stresses, along with key metabolic processes. An appropriate host enhances the overall adaptability, nutrient synthesis, and stress resistance of *M. oleifera* seedlings, all of which are essential for their growth, development, and survival.

## 1 Introduction

Root hemiparasitic plants have a wide range of uses, including as food, timber, and medicinal resources (Bell and Adams, 2011; Pignone and Hammer, 2016). However, because they rely on host plants for survival, cultivating them artificially presents some technical challenges (Pignone and Hammer, 2016; ArunKumar et al., 2022). Investigating the cultivation techniques for each economically valuable root hemiparasitic plant is both a fascinating and practically important endeavor. *Malania oleifera*, a monotypic species in the Olacaceae family, is an endangered tree endemic to China and native to the karst landscapes of southeastern Yunnan and western Guangxi provinces. The species was described scientifically in 1980 (Lee, 1980). In 1981, researchers discovered that the seed oil of *M. oleifera* is rich in nervonic acid (15-tetracosenic acid) (Ou, 1981). Nervonic acid plays a crucial role in human health, contributing to brain development and maintenance, improving memory, and delaying brain aging (Li Q. et al., 2019). The oil content of *M. oleifera* seed ranges between 48.3% and 67.9%, with nervonic acid comprising between 55.7% and 67.0% of the total fatty acids, making this species a valuable source of this nutrient (Ou, 1981; Tang et al., 2013; Wang et al., 2021). *M. oleifera* therefore stands out as a promising candidate for the discovery and development of nervonic acid resources. However, due to the scarcity of wild *M. oleifera* resources, cultivation would be the necessary path for its sustainable development and utilization.

Between the 1980s to about 2008, attempts to cultivate *M. oleifera* in the wild within its native habitat have yielded poor results (Liu, 1984; Huang et al., 2008), and the plants exhibited significant variation in individual growth. Some perished rapidly after a brief period of development, while others either grew slowly or not at all, and were susceptible to root diseases. Few individuals displayed robust growths, and overall, the survival rate was low, which was puzzling and which differed from the typical responses of other cultivated plants. Subsequently, researchers conducted afforestation experiments in four types of sites: open forests, shrubland, grasslands, and bare land. They found that the survival rates and growth of *M. oleifera* seedlings were highest in open forests, followed by shrubland, grasslands, and finally bare land. This phenomenon was attributed to the differences in growth environments caused by varying vegetation types (Lv et al., 2016). The unusual and seemingly unpredictable growth of *M. oleifera* after planting made effective cultivation challenging, and although the species has great economic value, this technical bottleneck in its development and utilization lies in the challenges of successful artificial cultivation.

By 2019, researchers had discovered that *M. oleifera* is a root hemiparasitic plant, a finding that offered new insights for the exploration of novel cultivation methods for the species (Li Y.P. et al., 2019, Li A.R. et al., 2019). Subsequent experiments were conducted to pair *M. oleifera* seedlings with various host plant species. While the seedlings successfully established parasitic relationships with the hosts, many of these host plants did not significantly enhance the growth of the seedlings (Li Y.P. et al., 2019; Chen et al., 2022). This outcome contrasts with the typical behavior observed in other root hemiparasitic plants, which often experience substantial growth benefits from their hosts (Chen et al., 2020; Thyroff et al., 2023). The specific conditions under which host plants can effectively promote the growth of *M. oleifera* seedlings therefore remain unclear.

In the absence of a host, the length of the normal growth period for root hemiparasitic plants depends on the characteristics of the plant itself and is also influenced by the growth environment. In the absence of a host, different soil nutrient levels have varying effects on the growth of different root hemiparasitic plant species (Kokla et al., 2022; Speetjens and Jacobs, 2023). When nutrients are artificially supplied, some root hemiparasitic plants can complete their entire life cycle in the absence of a host (Seel et al., 1993; Li et al., 2013). However, in some plants, seedling growth stagnates without a host even if nutrients are artificially supplied (Musselman, 1969; Těšitel et al., 2010). For *M. oleifera*, which is naturally distributed in karst regions, the extent to which soil conditions affect seedling growth it is still unknown.

The physiology of root hemiparasitic plants undergoes significant changes before and after a parasitic relationship with their hosts is established. Plant hormones play a crucial role in haustorium development, with changes in endogenous hormone levels contributing to this process. Through the haustorium, various substances, including secondary metabolites, RNAs, proteins, and nutrients, are transferred between parasitic plants and their hosts. These physiological changes are closely linked to the regulation of specific genes (Zhang et al., 2012; Shen et al., 2023; Ashapkin et al., 2023).

Transcriptomic analyses using RNA sequencing (RNA-seq) and *de novo* assembly have revealed the conservation of chlorophyll synthesis in root-parasitic Orobanchaceae (Wickett et al., 2011), as well as host-specific patterns of parasite gene expression at the interface between *Triphysaria versicolor* and its hosts (Honaas et al., 2013). The expression of haustorial genes differs in some hemiparasitic plants before and after establishing a parasitic relationship with the host (Zhang et al., 2015) as do levels of endogenous hormones levels and gene expression in the roots (Chen et al., 2021). Whole genome sequencing provides comprehensive background information for transcriptome sequencing, significantly enhancing the interpretation of transcriptomic data and enabling a more accurate understanding of the biological significance within the data. High-quality genomic data for *M. oleifera* have recently become available, and will enhance the analysis of its transcriptome data and biological functions (Yang et al., 2023). However, to date, a comprehensive understanding of the difference in gene expression differences in haustoria and roots is lacking, as is understanding of the intrinsic systemic connections between these differences and physiological changes. Moreover, the correlation between the changes at the micro level (gene expression differences and physiological changes) and the growth performance of the plants at the macro level needs further research.

Our preliminary experiments demonstrated that *M. oleifera* seedlings can establish a parasitic relationship with a fast-growing subshrub, *Tithonia diversifolia*. This host plant effectively promotes the growth of *M. oleifera* seedlings. Based on these findings, we will address the following questions: (1) Which factor is more effective in promoting the growth of *M. oleifera* seedlings: the host plant or soil conditions? (2) Is there a direct correlation between the biomass of the host plant and the biomass of attached *M. oleifera* seedlings? (3) What are the differences in gene expression in haustoria and roots when plants are grown under non-parasitic and parasitic conditions, and how do these physiological changes affect seedling growth? This knowledge will not only enhance our understanding of the growth patterns of root hemiparasitic tree species, but also aid in developing effective artificial cultivation methods for economically valuable but rare root hemiparasitic trees.

## 2 Materials and methods

### 2.1 Plant materials

In October 2021, we collected a substantial number of mature fruits from wild *M. oleifera* plants in Guangnan County, Yunnan. We removed the peel and cleaned the seeds, we then selected seeds of uniform size and sowed them in a sand bed at the Kunming Arboretum. In early July 2022, once the seeds in the sand bed had germinated and developed 5–6 true leaves, we transplanted them into planting bags measuring 12 cm in diameter and 32 cm in height. The seedlings were then transplanted into one of two different substrates: either a nutrient-rich substrate (Substrate I) consisting of a 1:1 ratio of soil and organic fertilizer (V/V), or a nutrient-poor substrate (Substrate II) consisting of a 1:1 ratio of fine sand and perlite (V/V). The nutrient content of each substrate type is presented in **Table 1**. Seedling on each substrate type were either given access to a host plant, or not. The four resulting treatments were named as follows: substrate I without a host: S1N, substrate I with a host: S1H, substrate II without a host: S2N, and substrate II with a host: S2H. Each treatment was replicated 30 times, yielding a total of 120 planting bags. *M. oleifera* seedlings were planted at the center of each bag. In the planting bags with host plants, a *T. diversifolia* stem cutting, measuring 25 cm in length and 1.5 cm in diameter, was inserted obliquely beneath the root of the *M. oleifera* seedling. Two-thirds of the *T. diversifolia* cutting was buried in the substrate, maintaining a distance of 3 cm between the cutting and the seedling stem on the surface of the substrate. Preliminary experiments showed that stem cuttings of *T. diversifolia* root easily and tend to survive. The plants were planted in July, and the stem cuttings of *T. diversifolia* took root in about 10 days; The *M. oleifera* seedlings had established a parasitic relationship with their hosts after approximately one month. These planting bags were placed in a greenhouse with 50% shading and allowed to grow for one year. The average temperature in the greenhouse was 24°C in July and 10°C in January, and had a relative humidity of between 60% and 80%. These greenhouse conditions closely resemble the climatic conditions of the natural distribution area of *M. oleifera*. The planting bags were arranged with each bag spaced 30 cm apart. During the one-year experiment, hand weeding was performed as needed, and watering was carried out appropriately based on the moisture conditions of the substrate throughout the different seasons.

**Table 1:** Nutrient content of the two planting substrates.

Substrate type	SOM (g/kg)	TN (g/kg)	TP (g/kg)	TK (g/kg)	HN (mg/kg)	AP (mg/kg)	AK (mg/kg)
Substrate I	132.50 ± 2.63	5.31 ± 0.14	2.52 ± 0.23	6.85 ± 0.10	340.17 ± 11.50	44.22 ± 8.51	251.17 ± 4.56
Substrate II	1.86 ± 0.57	0.07 ± 0.01	0.07 ± 0.02	2.50 ± 0.96	4.00 ± 0.89	1.47 ± 0.68	13.17 ± 2.14

After thoroughly mixing each substrate, six samples were randomly selected for measurement. SOM, soil organic matter; TN, total N; TP, total P; TK, total K; HN, hydrolysable N; AP, available P; AK, available K.

### 2.2 Monitoring growth data, sampling and harvesting

In early August and early October of 2022, as well as early January, April, June, and August of 2023, we measured the basal stem diameter, height, and leaf number of each *M. oleifera* seedling. Following the final growth measurements, the treatments S1N and S1H, that used the same substrate but exhibited significant differences in seedling growth, were selected for transcriptome and hormone analysis. Four types of samples were collected: roots from *M. oleifera* seedlings grown without a host (S1N_R) and their haustoria (S1N_H, as *M. oleifera* can form haustoria even in the absence of a host); as well as roots from seedlings grown with a host (S1H_R) and haustoria attached to the host (S1H_H). Each sample included three replicates, with each replicate consisting of 3 randomly selected individual roots or haustoria. All samples were immediately frozen in liquid nitrogen and stored at -80°C for future analysis. Root samples were used only for RNA sequencing, while haustorium samples were also used for hormone analysis.

In order to monitor the growth of the host and parasite plants, we next rinsed the substrate in each planting bag thoroughly with clean water. In bags containing host plants, we counted the number of haustoria formed by each *M. oleifera* seedling parasitizing the roots of *T. diversifolia*. The seedlings were then carefully separated from the host roots, ensuring that the root systems of the *M. oleifera* seedlings remained intact. We then used an electronic balance to measure the fresh weight of the aboveground biomass and the root biomass of each *M. oleifera* seedling, along with the fresh weight of the aboveground biomass of *T. diversifolia*. The biomass samples were placed in an oven at 60°C for 48 hours, after which we measured the dry weight of each sample.

### 2.3 RNA extraction and sequencing, and endogenous hormone measurement in haustoria

Total RNA was extracted from tissue samples using TRIzol Reagent (Ambion, Cat# 15596018) following the manufacturer’s protocol (For detailed methods, see **Supplementary Data Sheet 1**). Concentration and purity were measured using a NanoDrop spectrophotometer (Thermo Scientific NanoDrop 2000, Thermo Scientific, Waltham, Massachusetts, USA). RNA integrity was evaluated using RNA-specific agarose gel electrophoresis and the Agilent 2100 Bioanalyzer with the RNA 6000 Nano Kit (5067-1511, Agilent Technologies Inc., California, USA). Poly-A tail-containing mRNA was enriched from total RNA using Oligo(dT) magnetic beads, and the RNA was then sheared into fragments of approximately 300 bp using ion fragmentation. Using the RNA as a template, the first strand of cDNA was synthesized with random hexamer primers and reverse transcriptase, followed by the synthesis of the second cDNA strand using the first strand as a template. After library construction, PCR amplification was used to enrich the library fragments, and library quality was assessed with the Agilent 2100 Bioanalyzer. Paired-end (PE) sequencing was subsequently performed on the libraries using next-generation sequencing (NGS) technology on the Illumina platform. The raw sequencing data was filtered to generate high-quality clean data, which were then aligned to the reference genome of *M. oleifera* (Yang et al., 2023). Gene expression levels were calculated based on the alignment results, followed by differential expression analysis, enrichment analysis, and clustering analysis of the samples.

Samples were prepared for LC-MS/MS analysis as follows. 40 mg of lyophilized sample was weighed into a 2 mL brown centrifuge tube, 1 mL each of methanol and mixed internal standard stock solution was accurately measured and added to the sample. Samples were sonicated for 10 min, then transferred to a metal bath and shaken. After 4 hours, samples were centrifuged at 12000 rpm for 10 min at 4 °C, and the entire supernatant was removed. 0.5 mL methanol was added to the remaining residue and samples were further shaken for 2 h in a metal bath. The extracts were then centrifuged through a 0.22 μm filter membrane and placed in the injection vial for LC-MS/MS analysis (Li et al., 2016).

### 2.4 qRT-PCR verification of DEGS

A total of 16 differentially expressed genes were selected for expression analysis, including 6 genes specifically identified from haustoria, 7 genes specifically identified from roots, and 3 genes selected from both haustoria and roots. RNA extraction and complementary DNA (cDNA) synthesis were performed following the protocols described in Section 2.3. The forward and reverse primers were designed by Primer Premier (version 6.0, Premier Biosoft Inc., CA, USA). The reaction was performed using the Power qPCR PreMix (Genecopoeia) in a 96-Well PCR plate (PCR-96-FLT-C, Axygen). The reaction conditions were established according to the manufacturer’s instructions, with each reaction performed in triplicate. The *actb* was employed as the reference gene. The 2^−ΔΔCt^ method was employed to calculate relative expression levels. The detailed experimental procedures and specific primers are listed in **Supplementary Data Sheet 1**.

### 2.5 Statistical analysis

The cumulative growth from each monitoring event and the harvested biomass of *M. oleifera* seedlings were analyzed using ANOVA (One-way analysis of variance). The annual cumulative growth of each *M. oleifera* seedling, the harvested biomass, the number of haustoria connected to the host, and the aboveground biomass of *T. diversifolia* (only for plants with a host) were analyzed for correlations using Pearson correlation analysis. The hormone content in the parasitic haustoria was analyzed using an independent samples t-test. All statistical analyses were performed using the R statistical package.

We performed statistical analysis on the raw data (Raw Data) of each sample, including sample name, Q30, percentage of ambiguous bases, and Q20(%) and Q30(%). Sequencing data, including adapter-contaminated and low-quality reads, were then filtered using Fastp to remove 3’ adapter sequences and reads with an average quality score below Q20 were discarded. The filtered reads were aligned to the reference genome (GCF_029873635.1_ASM2987363v1_genomic.fna) using the HISAT2 software and the mapping information was calculated.

We used HTSeq to count the number of reads aligned to each gene, which served as the raw gene expression values. FPKM (Fragments Per Kilo bases per Million fragments) was used to normalize the expression levels, and genes with FPKM > 1 were considered to be expressed. We performed differential expression analysis using DESeq, with the criteria for selecting differentially expressed genes set as |log2FoldChange| > 1 and a significance threshold of P-value < 0.05. Differentially expressed genes (DEGs) were analyzed separately using Gene Ontology (GO) and the Kyoto Encyclopedia of Genes and Genomes (KEGG) database, and were annotated and enriched to obtain functional analyses and pathway results for DEGs.

## 3 Results

### 3.1 The growth dynamics of *M. oleifera* under different planting scenarios

**Figure 1:**
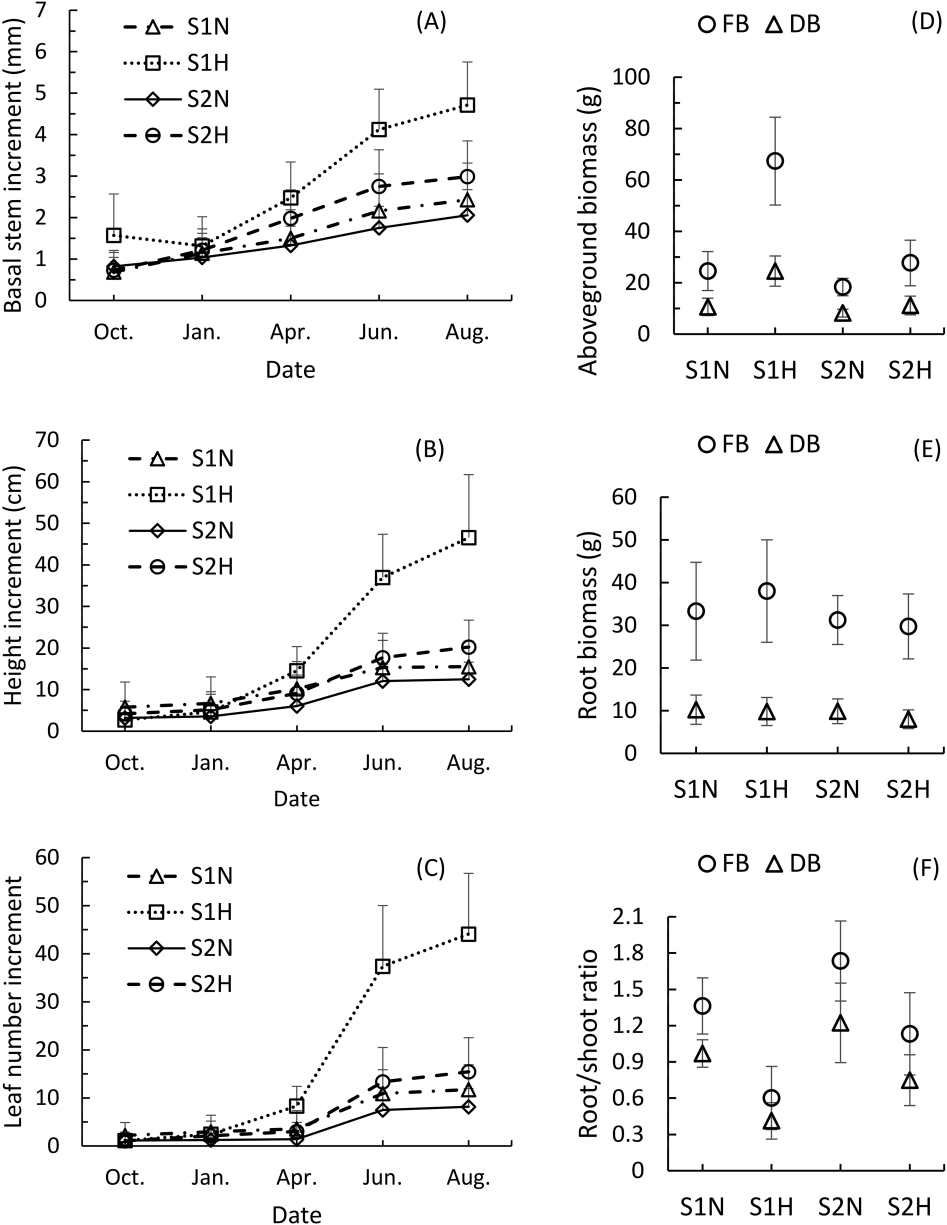
Comparison of the growth dynamics and biomass of *Malania oleifera* seedlings under four planting treatments (mean ± SD, n=30). **(A)** Annual growth dynamics of basal stem diameter. **(B)** Annual growth dynamics of plant height. **(C)** Annual growth dynamics of plant leaf number. **(D)** Aboveground fresh and dry biomass of plants for a whole year. **(E)** Root fresh biomass and dry biomass of plants for a whole year. **(F)** Annual root-to-shoot ratio analysis of plant growth. FB, Fresh biomass; DB, Dry biomass. S1N: nutrient-rich substrate without host; S1H, nutrient-rich substrate with host; S2N, nutrient poor substrate without host; S2H, nutrient-poor substrate with host.

The cumulative growth of the monitored growth indicators (increase in stem diameter, plant height, and leaf number) showed similar growth trends across the four planting methods (**Figures 1A-C**). The fastest-growing plants were all in treatment S1H, and showed significant growth differences as early as April of the second year. The increases in stem diameter, plant height, and leaf number were all significantly greater than those of plants subjected to the other three treatments (P<0.01). By August of the second year, the growth differences had become more pronounced, with S1H plants showing highly significant differences in growth compared to plants subjected to the other three treatments (S1N, S2N, S2H) (P<0.001).

The second fastest-growing group was S2H. By August of the second year, the increases in stem diameter, plant height, and leaf number of plants in treatment group S2H were significantly greater than those of plants subjected to treatments S1N (P<0.05) and S2N (P<0.01). S1N plants grew faster than those in the S2N group, but by August of the second year, the differences in these indicators were not significant (P>0.05). These findings indicate that selecting an effective host plant is conducive to promoting the growth of *M. oleifera* seedlings, and, while favorable soil conditions can also directly enhance seedling growth, though the effect is not significant.

The fresh and dry weights of the aboveground biomass of *M. oleifera* seedlings across the four treatments showed similar trends to their growth patterns (**Figure 1D**). S1H plants had the highest aboveground fresh and dry biomass (67.32 ± 17.09g, 24.53 ± 5.88g), followed by plants in groups S2H (27.68 ± 8.87g, 11.14 ± 3.63g), S1N (24.55 ± 7.59g, 10.59 ± 3.38g), and S2N (18.33 ± 3.39g, 8.16 ± 1.51g). The fresh and dry aboveground biomass of plants under treatment S1H was significantly different from plants under the other three treatments (P<0.001). The fresh and dry aboveground biomass of S2H plants were significantly greater than those of S2N plants (P<0.01), but the difference between S2H and S1N plants was not significant (P>0.05). The differences in fresh and dry aboveground biomass between plants subjected to treatments S1N and S2N were significant (P<0.05).

The root biomass showed a different pattern from the aboveground biomass (**Figure 1E**). Plants in group S1H had the highest fresh root weight (38.01 ± 12.00g), followed by those in S1N (33.29 ± 11.45g), S2N (31.23 ± 5.73g), and S2H (29.73 ± 7.60g). Plants subjected to treatment S1N had the highest dry root weight (10.23 ± 3.43g), followed by those in treatment groups S2N (9.86 ± 2.90g), S1H (9.80 ± 3.29g), and S2H (7.99 ± 2.20g). Significant differences in fresh root biomass were only found between S1H and S2N, and S1H and S2H (P<0.01), while no significant differences were observed between any other pairs (P>0.05). The only significant differences in dry root biomass were found between plants in the S2H treatment group and those subjected to the other three treatments (P<0.05).

However, the root-to-shoot ratio exhibited the opposite trend compared to aboveground growth (**Figure 1F**). Plants subjected to treatment S1H had the smallest root-to-shoot ratio, with fresh and dry values of 0.60 ± 0.26 and 0.41 ± 0.15, respectively. In comparison, S2H plants had root-to-shoot ratios of 1.13 ± 0.34 and 0.75 ± 0.21, S1N plants had 1.36 ± 0.23 and 0.97 ± 0.11, and S2N had 1.73 ± 0.33 and 1.22 ± 0.33. The aboveground biomass growth of *M. oleifera* seedlings parasitizing *T. diversifolia* increased significantly compared with that of seedlings without a host plant, while root biomass growth lagged, resulting in an imbalance between aboveground and belowground development.

**Figure 2:**
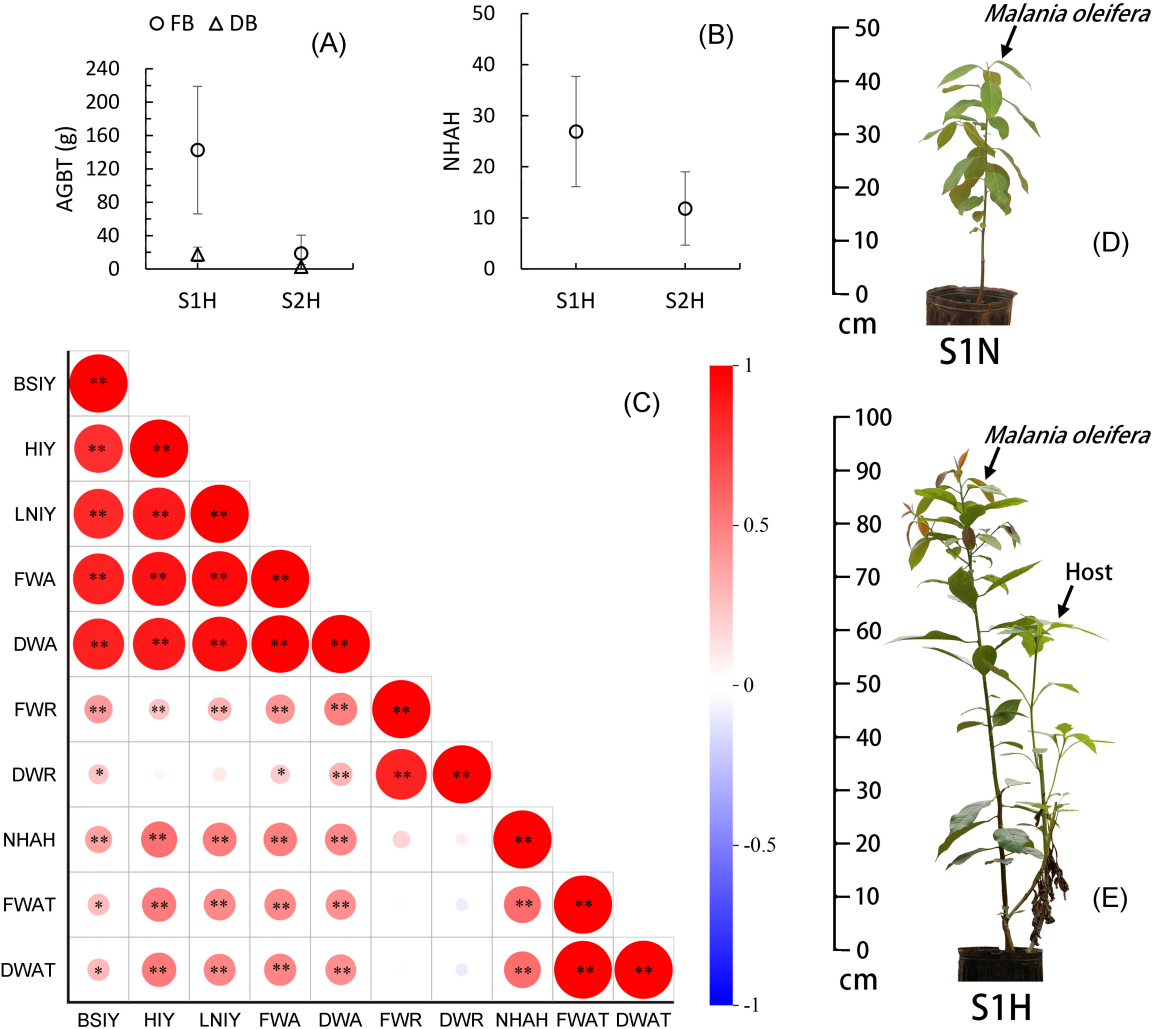
Correlation between *Malania oleifera* seedlings and host growth. **(A)** Aboveground biomass of *T. diversifolia* (AGBT) (mean ± SD, n=30). **(B)** the number of *M. oleifera* haustoria attached to the host (NHAH) (mean ± SD, n=30). **(C)** AGBT, NHAH and Seedling Growth Correlations in *M. oleifera* (n=60). **(D, E)** Growth comparison of representative plant under S1N and S1H treatments (To better display the parasitic plant, some branches of the host were removed). FB, Fresh biomass; DB, Dry biomass; BSIY, Basal stem increment for a whole year; HIY, Height increment for a whole year; LNIY, Leaf number increment for a whole year; FWA, Fresh weight of aboveground biomass; DWA, Dry weight of aboveground biomass; FWR, Fresh weight of root biomass; DWR, Dry weights of root biomass; FWAT, Fresh weight of aboveground biomass of *T. diversifolia*; DWAT, Dry weight of aboveground biomass of *T. diversifolia*. *Significance at P < 0.05, **significance at P < 0.01.

Under different soil conditions, the growth of the host plant *T. diversifolia* showed highly significant differences (P<0.01). The aboveground biomass of *T. diversifolia* in the nutrient-rich S1H treatment group had fresh and dry weights of 142.46 ± 76.46g and 17.43 ± 8.85g, respectively, while those in the nutrient-poor S2H treatment group had fresh and dry weights of 18.61 ± 2.20g and 2.44 ± 2.87g, respectively (**Figure 2A**). The number of haustoria of *M. oleifera* parasitizing the roots of *T. diversifolia* also showed highly significant differences (P<0.01) across different substrates, with 26.90 ± 10.80 haustoria produced by plants subjected to the S1H treatment, but only 11.83 ± 7.18 in the S2H treatment group (**Figure 2B**). Further correlation analysis revealed that the aboveground biomass (fresh and dry weight) of *T. diversifolia* was significantly positively correlated with *M. oleifera* basal stem increment (P<0.05), height increment (P<0.01), leaf number increment (P<0.01), fresh and dry aboveground biomass (P<0.01), and the number of parasitic haustoria (P<0.01), but showed no correlation with its root biomass (fresh and dry weight). Additionally, the number of haustoria was highly significantly positively correlated (P<0.01) with basal stem increment, height increment, leaf number increment, and the fresh and dry aboveground biomass of *M. oleifera* (P<0.01) (**Figure 2C**). Clearly, the growth of the host plant *T. diversifolia* significantly promoted the aboveground biomass growth of *M. oleifera*, while having little effect on root growth.

### 3.2 Quality assessment of transcriptome sequencing data from haustoria and roots

82.2 G of raw data were obtained from transcriptome sequencing of twelve samples (**Supplementary Table S1**). The raw data for all twelve samples had a Q20 quality score of 98.05% and a Q30 quality score of 94.28%. The GC content of the raw reads ranged from 43.39% to 46.22%. The clean data mapping rates to the *M. oleifera* genome were more than 93.66%, except for sample S1H-R06 (73.56%). The haustorium and root transcriptome sequencing data with high quality and mapping ratios were used for subsequent RAN analysis. The correlation analysis of gene expression levels between biological replicates showed that the Pearson correlation coefficients were all greater than 0.89, except for the value (0.77) between sample S1N_H01 and S1N_H03 (**Supplementary Table S2**).

### 3.3 Differential gene expression in *M. oleifera* haustoria and roots under non-parasitic and parasitic conditions

**Figure 3:**
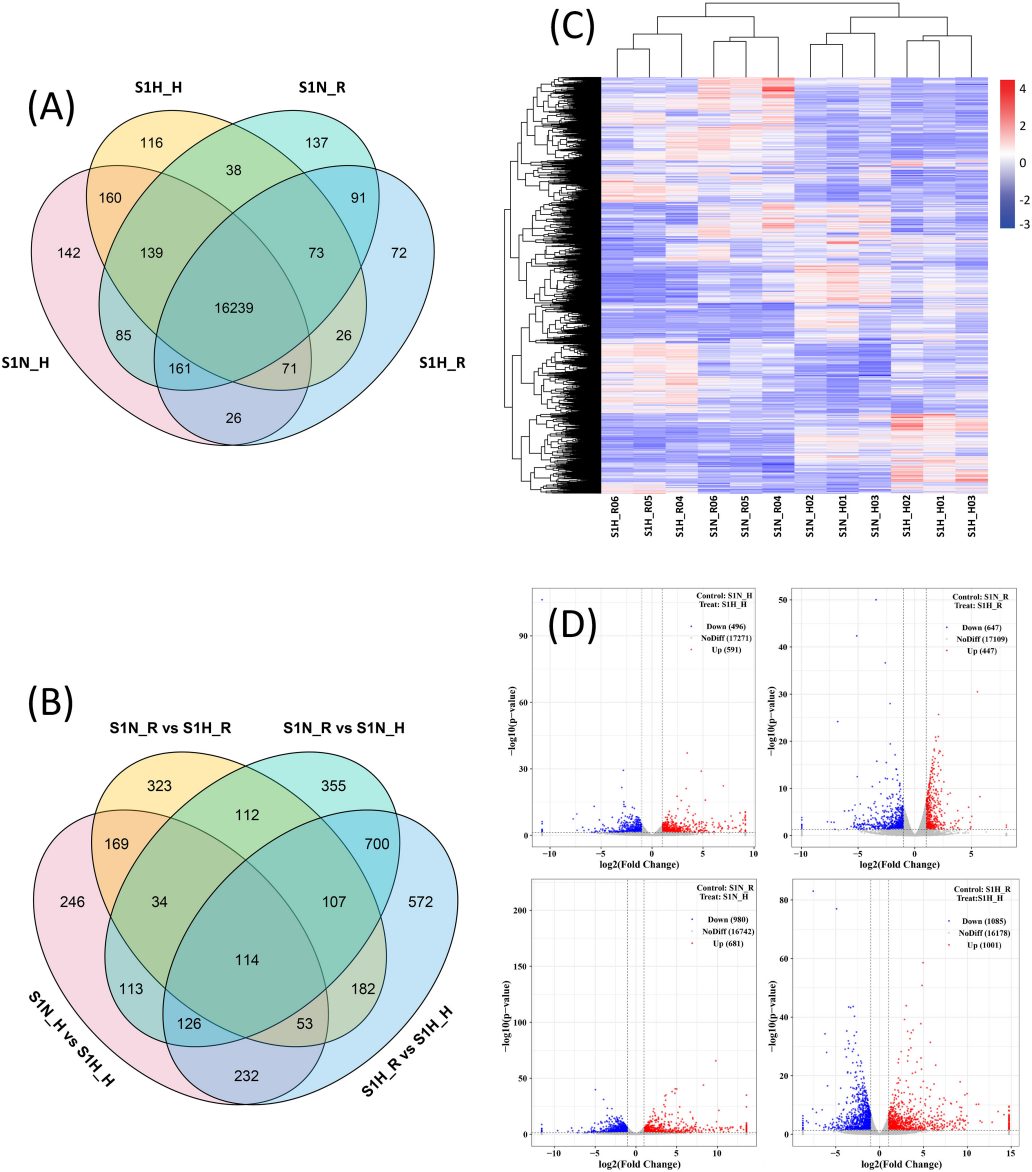
Gene expression in haustoria and roots of *Malania oleifera* under different planting treatments. **(A)** Number of expressed genes in *M. oleifera* haustoria and roots. **(B)** Number of DEGs. **(C)** The DEGs cluster heat map. **(D)** Volcano plot of DEGs.

17023 (S1N_H), 16963 (S1N_R), 16862 (S1H_H), and 16759 (S1H_R) genes (expressed in all three replicates) were expressed in the haustoria and roots of plants in the S1N and S1H treatment groups, respectively (**Figure 3A**). The numbers of DEGs for S1N_H vs S1H_H, S1N_R vs S1H_R, S1N_R vs S1N_H and S1H_R vs S1H_H were 1087, 1094, 1661 and 2086, respectively (**Figure 3B**). There were 591, 447, 681 and 1001 up-regulated DEGs between S1N_H vs S1H_H, S1N_R vs S1H_R, S1N_R vs S1N_H and S1H_R vs S1H_H, respectively, and 496, 647, 980 and 1085 down-regulated DEGs, respectively (**Figure 3D**). The cluster analysis demonstrated that the differences in gene expression were significant among these four treatment groups (**Figure 3C**).

### 3.4 Functional changes in the haustoria of *M. oleifera* seedlings between non-parasitic and parasitic states

GO annotations and KEGG pathways analyses were used to analyze changes in haustorium function in non-parasitic and parasitic *M. oleifera* plants. The number of GO terms that were significantly enriched in up-regulated DEGs in the haustoria of *M. oleifera* plants without versus with a host (S1N_H vs S1H_H), was much higher than in down-regulated DEGs. A total of 140 GO terms were associated with up-regulated DEGs (FDR < 0.01), while only 12 GO terms were linked to down-regulated DEGs. Most GO terms for up-regulated DEGs were related to biological processes, including the regulation of hormone metabolism and biosynthesis (e.g., jasmonic acid and salicylic acid), response to ozone, antibiotic biosynthesis, and lipid metabolism and biosynthesis (**Supplementary Table S3**). In contrast, the down-regulated DEGs were mainly enriched in functions associated with the cell periphery, cell wall, and external encapsulating structure.

**Figure 4:**
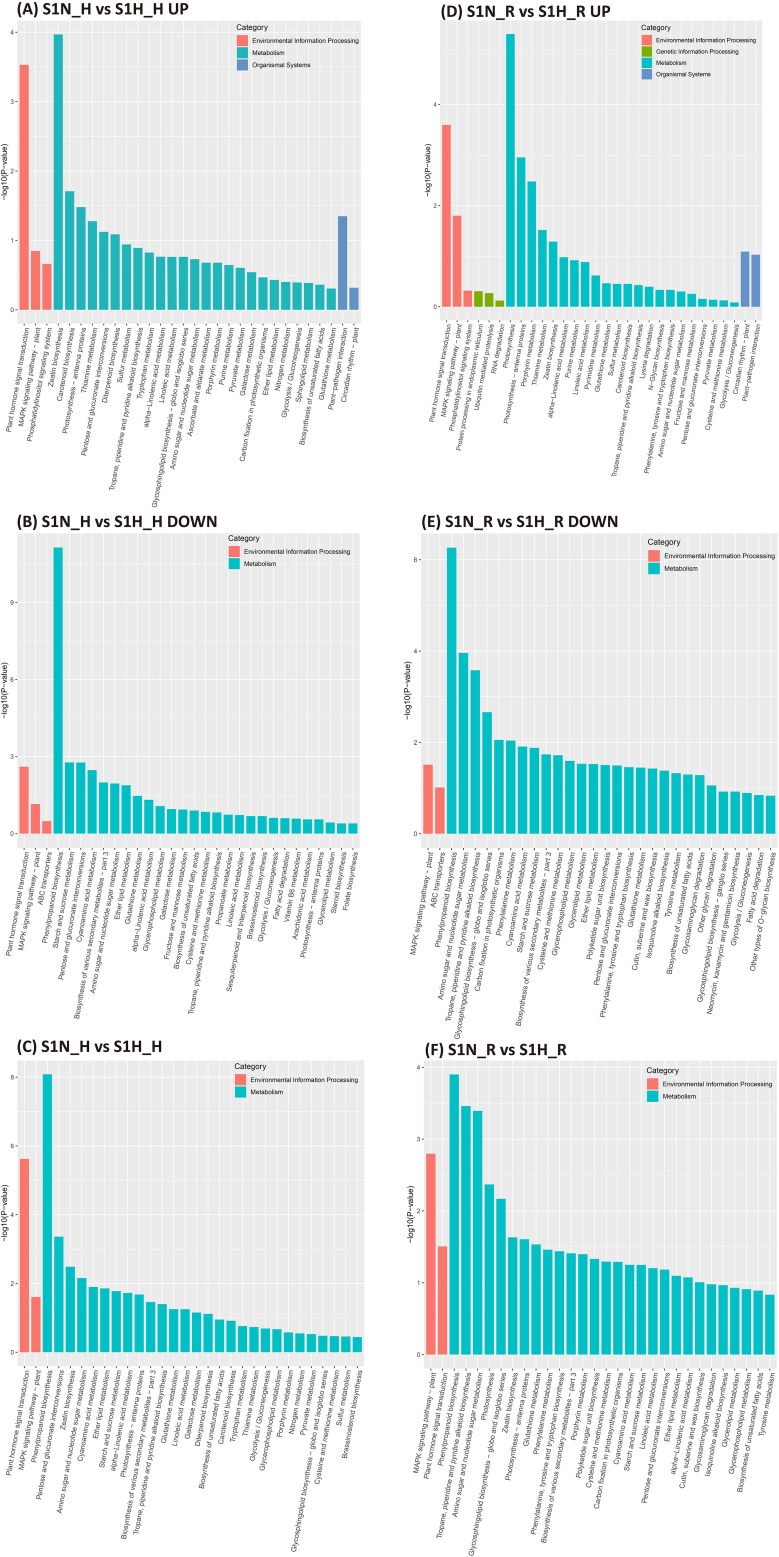
KEGG enrichment analysis in *Malania oleifera* haustoria and roots in the absence and presence of the host plant. **(A)** Up-regulated genes in S1N_H vs S1H_H. **(B)** Down-regulated genes in S1N_H vs S1H_H. **(C)** DEGs in S1N_H vs S1H_H. **(D)** Up-regulated genes in S1N_R vs S1H_R. **(E)** Down-regulated genes in S1N_R vs S1H_R. **(F)** DEGs in S1N_R vs S1H_R. S1N, nutrient-rich substrate without host; S1H, nutrient-rich substrate with host.

The KEGG pathways identified in the DEGs in the haustoria of *M. oleifera* plants without versus with a host (S1N-H vs S1H-H) were mainly associated with the biosynthesis of other secondary metabolites, signal transduction, carbohydrate metabolism, metabolism of terpenoids and polyketides, metabolism of other amino acids, lipid metabolism and energy metabolism (**Figure 4C**). The key functions of these identified up-regulated DEGs were enriched in the pathways of Zeatin biosynthesis, plant hormone signal transduction, carotenoid biosynthesis, photosysthesis-antenna proteins and plant-pathogen interactions (**Figure 4A**). The down-regulated DEGs were mainly enriched in the pathways phenylpropanoid biosynthesis, starch and sucrose metabolism, pentose and glucuronate interconversion, plant hormone signal transduction and cyanoamino acid metabolism (**Figure 4B**).

The results of the GO and KEGG analyses suggest that when the *M. oleifera* haustorium is connected to a host plant, resistance and adaptability of the hemiparasite are enhanced through regulation of hormone signaling, secondary metabolism, and activation of defense-related pathways. This regulatory mechanism involves the metabolism of various hormones (such as jasmonic acid and salicylic acid), the synthesis of secondary metabolites, protection against antioxidants, and adjustments in cell structure, enabling the plant to effectively respond to a range of biotic and abiotic stresses from the external environment.

### 3.5 Functional changes in the roots of *M. oleifera* seedlings in non-parasitic and parasitic states

GO annotations and KEGG pathways analyses were also used to analyze changes in root function between plants in non-parasitic and parasitic states. Eighty-two GO terms were associated with up-regulated DEGs (FDR < 0.01), and 42 GO terms with down-regulated DEGs (**Supplementary Table S3**), in *M. oleifera* roots under both non-parasitic and parasitic conditions (S1N_R vs S1H_R). Similar to haustorial DEGs, the GO terms related to up-regulated DEGs in roots were primarily involved in biological processes. The key functions of these up-regulated DEGs were enriched in responses to chitin, organonitrogen and oxygen-containing compounds, drug, chemical, other organisms, internal and external biotic stimuli, organic substances, stress, as well as in transcription regulator activity and DNA-binding transcription factor activity. In contrast, the down-regulated DEGs were mainly enriched in functions related to the cell periphery, cell wall, external encapsulating structure, enzymatic activity (including oxidoreductase, transferase, glucosyltransferase and quercetin glucosyltransferase), saponin metabolism and biosynthesis, responses to starvation, water and fluid transport.

The KEGG pathways for S1N-R vs S1H-R DEGs of roots were mainly associated with biosynthesis of other secondary metabolites, carbohydrate metabolism, signal transduction, energy metabolism and metabolism of terpenoids and polyketides (**Figure 4F**). The key functions of these up-regulated DEGs in S1N-R vs S1H-R were enriched in the pathways of photosynthesis, plant hormone signal transduction, porphyrin metabolism, the MAPK signaling pathway and thiamine metabolism (**Figure 4D**), and the down-regulated DEGs were mainly enriched in the pathways of phenylpropanoid biosynthesis, amino sugar and nucleotide sugar metabolism, tropane piperidine and pyridine alkaloid biosynthesis, glycosphingolipid biosynthesis (**Figure 4E** **).**

The GO and KEGG analysis data collectively indicate that the roots of *M. oleifera* seedlings, when associated with a host, actively participate in responses to environmental stresses, pathogen defense, biosynthesis of protective secondary metabolites, hormone-mediated signaling, and maintenance of cellular homeostasis. This highlights the complex regulatory networks in *M. oleifera* that balance growth, defense, and adaptation under stress conditions.

### 3.6 Functional comparison of the root systems of non-parasitic and parasitic *M. oleifera* seedlings

To investigate the impact of parasitism on the entire root system of *M. oleifera* seedlings, we compared the functional changes in DEGs (both in haustorial organs and roots) under non-parasitic conditions (S1N_R vs S1N_H) and parasitic conditions (S1H_R vs S1H_H). In the absence of parasitism, the DEGs between haustoria and roots (S1N_R vs S1N_H) were predominantly associated with basic metabolic and defense-related functions of the plant (**Supplementary Table S4**). These include processes such as cell wall biosynthesis and tissue development (e.g., GO:0005618 cell wall, GO:0009505 plant-type cell wall, GO:0071554 cell wall organization or biogenesis, GO:0099402 plant organ development, ko00940 phenylpropanoid biosynthesis, ko04075 plant hormone signal transduction), as well as hormone metabolism and fundamental responses to various stimuli under abiotic stress conditions (GO:0042445 hormone metabolic process, GO:0009725 response to hormone, GO:0010200 response to chitin, GO:0001101 response to acid chemical, GO:0050896 response to stimulus, ko04075 plant hormone signal transduction, ko00905 brassinosteroid biosynthesis, ko04016 MAPK signaling pathway), and core metabolic processes (GO:0005886 plasma membrane, GO:0006811 ion transport, ko00040 pentose and glucuronate interconversions, ko00500 starch and sucrose metabolism, ko00910 nitrogen metabolism, ko00270 cysteine and methionine metabolism, ko01040 biosynthesis of unsaturated fatty acids).

Under parasitic conditions, the DEGs between haustoria and roots (S1H_R vs. S1H_H) not only support basic growth functions but also specifically enhance defense responses and adaptation to biotic stress (**Supplementary Table S5**). This is achieved by regulating specific hormones (GO:0080140 regulation of jasmonic acid metabolic process, GO:0080141 regulation of jasmonic acid biosynthetic process, GO:0080142 regulation of salicylic acid biosynthetic process, GO:0010337 regulation of salicylic acid metabolic process, ko04075 plant hormone signal transduction) and by strengthening defense-related metabolic pathways (GO:0051707 response to other organisms, GO:0043207 response to external biotic stimulus, GO:0009607 response to biotic stimulus, ko00950 isoquinoline alkaloid biosynthesis, ko04626 plant-pathogen interaction) to counter insect or pathogen invasion. In summary, the root systems of *M. oleifera* seedlings without hosts sustains only the basic functions necessary for plant structure and growth. In contrast, in plants with a suitable host, the root systems significantly enhance the precise regulation of defense and stress responses, illustrating a more dynamic and adaptive response mechanism in plants under complex environmental conditions.

### 3.7 Changes of hormone content in haustoria and KEGG enrichment

**Figure 5:**
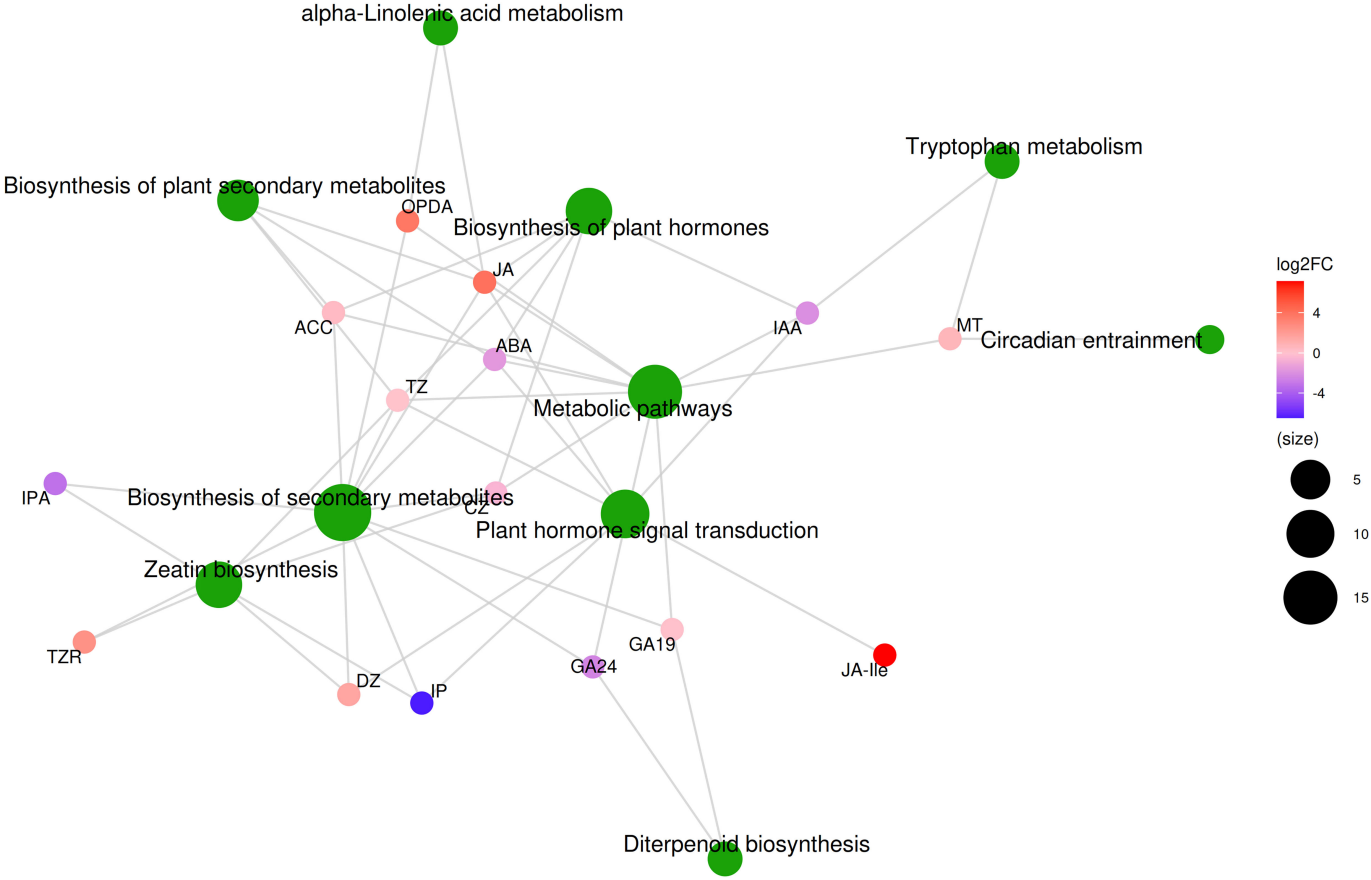
KEGG metabolic compound molecular network diagram.

We found significant differences in the hormone content of non-parasitic and parasitic *M. oleifera* haustoria (**Supplementary Table S6**). Of the 23 hormones detected in the haustoria, 6 showed significant differences (P < 0.05), while 3 displayed marginal differences (P = 0.089, P = 0.072, P = 0.061). In the parasitic haustoria, the contents of JA-Ile, JA, SAG, TZR, ICAId, ACC increased by 145.9 times, 16.3 times, 12.6 times, 5.6 times, 2.3 times and 1.3 times, respectively, while the contents of GA24, IAA and ABA decreased by 5.6 times, 4.2 times and 3.2 times, respectively. The levels of the 23 hormones detected in the haustoria showed varying degrees of correlation with the differential expression of 289 genes in the haustoria and 300 genes in the roots (**Supplementary Figure S1**). Metabolite pathway analysis showed that there were 15 hormones that affected KEGG pathways, with the main pathways affected being biosynthesis of plant secondary metabolites, biosynthesis of plant hormones, metabolic pathways, biosynthesis of secondary metabolites, plant hormone signal transduction, and zeatin biosynthesis (**Figure 5**). This suggests that during the development of *M. oleifera*, the hormone levels in haustoria parasitizing host plants undergo significant changes. These hormonal fluctuations influence various metabolic processes in the *M. oleifera*, ultimately impacting its growth.

### 3.8 Validation of DEGs using qRT-PCR

**Figure 6:**
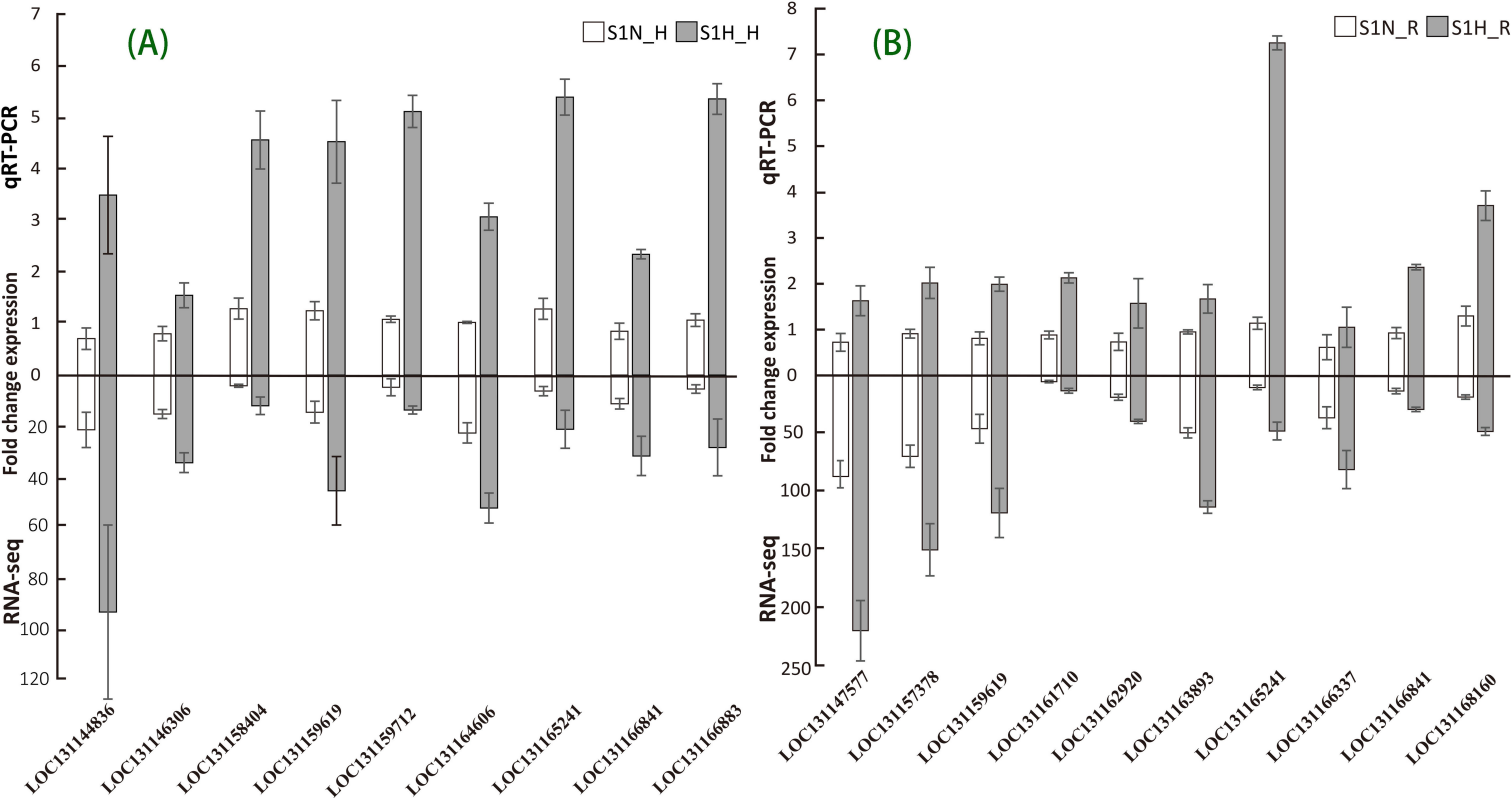
Comparison of the expressions levels of DEGs determined with RNA-Seq and qRT-PCR (Expression of the target DEGs was normalized to that of the β-actin gene). **(A)** Verification of DEGs in *M. oleifera* haustoria. **(B)** Verification of DEGs in *M. oleifera* roots.

To ensure the accuracy and reliability of the sequencing data under both non-parasitic and parasitic growth conditions, we selected 16 differentially expressed genes (DEGs), including 6 related to haustoria, 7 related to roots, and 3 shared by both tissues, for expression validation through quantitative real-time PCR (qRT-PCR). As shown in **Figure 6**, the qRT-PCR and RNA-Seq results were consistent. These verified genes involve abscisic acid receptor PYL9 (LOC131166841), auxin response factor (LOC131165241), transcription factor MYC2-like (LOC131163893), WRKY transcription factor WRKY24-like (LOC131147577), protein TIFY 9 (LOC131144836), two-component response regulator (LOC131164606), WRKY transcription factor (LOC131166883), and calmodulin-like protein (LOC131158404), which are closely related to the changes in the main physiological activities of *M. oleifera* under parasitic conditions.

## 4 Discussion

### 4.1 The impact of the host on the growth of *M. oleifera* seedlings

In the early stages of growth, root hemiparasitic plants can maintain a normal growth period for some time by absorbing nutrients stored in their seeds or by directly absorbing nutrients from the soil (Musselman, 1969; Seel et al., 1993; Těšitel et al., 2010; Li et al., 2013). *M. oleifera* seeds are relatively large, with a diameter of 2–5 cm, and are rich in oils (Wang et al., 2021), and after germination, these abundant nutrients are transferred to the seedling. Due to this nutrient reserve, seedlings grow vigorously in their first year, after which growth gradually weakens until it eventually stagnates. During this process, the concentrations of N, P, and K in most plant tissues, along with starch granule content, progressively decrease. Some individuals with weakened growth can survive for more than three years (Chen et al., 2024). Our study shows that *M. oleifera* seedlings grown in nutrient-rich substrate without a host (S1N treament) does not exhibit significant differences in increments of basal stem diameter, height, or leaf number compared to plants grown in nutrient-poor substrate (S2N treatment) (P > 0.05). This suggests that favorable soil conditions alone have limited effects on the growth of *M. oleifera*. In the presence of a host, however, *M. oleifera* growth is significantly promoted. Plants cultivated under the S1H treatment (nutrient rich substrate and in the presence of a host) show highly significant differences in basal stem diameter, height, and leaf number compared to the other three treatments (P < 0.001). When a host is present, nutrient-poor substrates (S2H treatment) promote *M. oleifera* seedling growth more effectively than do nutrient-rich substrates in the absence of a host (S1N treatment). The two experimental substrates (Substrate I: soil + organic fertilizer; Substrate II: fine sand + perlite) differed significantly not only in nutrient content, but also potentially in texture, water-holding capacity, microbiota, and possibly other aspects. However, despite these substantial variations in edaphic properties, the growth parameters of *M. oleifera* seedlings cultivated without host plants showed no statistically significant differences between the two substrates (P>0.05 for S1N vs S2N treatments). This suggests that soil conditions alone have a limited impact on seedling development. Instead, these findings further emphasize the critical role of a host plant in promoting the normal growth of *M. oleifera* seedlings.

Faster-growing host plants are known to more effectively promote the growth of parasitic plants than slow-growing hosts (Hautier et al., 2010). In our study, the growth of *M. oleifera* seedlings was closely connected to the growth of the host plant. The aboveground biomass (fresh and dry weight) of the host *T. diversifolia* was highly significantly correlated with the height increase, leaf number, and aboveground biomass (fresh and dry weight) of *M. oleifera* seedlings (P < 0.01) and significantly correlated with basal stem diameter increase (P < 0.05; **Figure 2C**). This indicates that the rapid growth of *T. diversifolia* effectively promoted the rapid growth of *M. oleifera*. Sufficient nutrient acquisition from the host is essential to ensure the rapid growth of the parasitic plant. Previous studies have used tree seedlings of the species *Pinus armandii*, *Pistacia weinmannifolia*, and *Alnus ferdinandi-coburgii*, as well as herbaceous plants including *Solanum tuberosum*, *Chlorophytum comosum*, and *Artemisia argyi*, as host plants for *M. oleifera* seedlings, but none of these hosts significantly enhanced the growth of *M. oleifera* seedlings (Li Q. et al., 2019; Xiong et al., 2024). This may be due to the relatively slow growth rate of these hosts, which limits their ability to rapidly promote *M. oleifera* seedling growth. Furthermore, because parasitic plants draw nutrients and water from their host, they can also weaken the growth of the host and can even lead to host death (ArunKumar et al., 2022). Although root hemiparasitic plants are not highly specific in host selection, an ideal host should exhibit fast growth and strong adaptability. Given its rapid growth, tolerance to poor soils, and resilience, *T. diversifolia* appears to meet these criteria, which likely explains the significant growth improvement observed in parasitic *M. oleifera* in this study (S1H treatment).

The host plant used in our experiments, *T. diversifolia*, has a significantly greater effect on the aboveground growth of *M. oleifera* than on its root growth. When comparing the S1N and S1H treatments, the differences in aboveground biomass (fresh and dry weight) of the *M. oleifera* seedlings were extremely significant (P < 0.001), while the differences in root biomass were not significant (P > 0.05; **Figures 1D, E**). Moreover, the aboveground biomass of *T. diversifolia* (both fresh and dry weight) was not correlated with the root biomass of *M. oleifera* (P > 0.05; **Figure 2C**). This means that the growth of the above- and belowground parts of *M. oleifera* seedlings in a parasitic state is unbalanced. This uneven growth prevents *M. oleifera* from rapidly developing a large root system, which would enable it to obtain more water and nutrients, as well as from quickly establishing other parasitic connections with other potential host plants, and therefore leads to the seedlings being extremely vulnerable. If the host plant weakens or dies due to parasitism, the *M. oleifera* will likewise weaken or die. In our previous field studies, we found that *M. oleifera* seedlings that initially exhibited good growth often later experienced stagnation or even death, likely due to this unbalanced growth (Personal communication).

### 4.2 The role of haustoria and roots in supporting the growth of *M. oleifera* under parasitic conditions

Haustoria are specialized structures in parasitic plants that form a physiological bridge with the host, penetrating its tissues and creating a vascular connection to absorb water, nutrients, RNA, proteins, and hormones (Kokla et al., 2022; Shen et al., 2023). The physiology of the haustoria varies significantly before and after the establishment of the parasite-host association (Chen et al., 2021). Biological processes in *M. oleifera* haustoria were significantly enhanced after establishment of the parasite-host association, and of the top 30 GO terms enriched in up-regulated genes, 29 were related to biological processes. (**Supplementary Table S3**). GO terms enrichment and KEGG pathway analysis of haustoria up-regulated genes (S1N_H vs S1H_H) revealed that the haustoria exhibited enhanced hormone signaling and photosynthesis regulation, responses to pathogens and environmental stresses, secondary metabolism, antioxidant defense, and metabolic pathway regulation. This indicates that the plants are actively adapting to both internal and external growth environments, optimizing their basal metabolism, and strengthening their defense mechanisms and adaptability. Overall, these gene enrichments reflect the plant’s ability to coordinate multiple metabolic pathways and signal transduction mechanisms to maintain survival and adaptability under various stresses.

GO term annotations and KEGG pathway analyses indicated that, following the establishment of *M. oleifera*-host associations, the physiology of the roots was also enhanced in response to biotic and abiotic stresses, as well as in photosynthetic activity, energy production, and secondary metabolism. Additionally, the capacities for basic metabolism and material conversion were improved, including the metabolism of nitrogenous compounds, carbohydrates, and lipids. This indicates that the overall ability of *M. oleifera* to adapt to its environment, utilize nutrients, synthesize essential compounds, and maintain vital functions significantly increased following establishment of the association with the host plant. Such enhancements are crucial for the plant’s growth, development, and resilience to environmental stress.

The hormone levels in the haustoria of *M. oleifera* seedlings attached to a host exhibited significant changes, with 13 of the top 30 most enriched GO annotations (S1N-H vs. S1H-H) for up-regulated genes linked to hormone-related physiological activities. Such significant fluctuations in hormone levels within the haustoria upon attaching to the host have been observed in various hemiparasitic plants (Ashapkin et al., 2023). For example, following contact with the host, the expression levels of genes involved in the synthesis and regulation of hormones such as IAA, cytokinins, GAs, ABA, ACC, and JA changed significantly in the haustoria of the root hemiparasite *Santalum album* (Zhang et al., 2015). Our research further confirmed that the levels of these plant hormones in the haustoria of *M. oleifera* exhibited significant increases or decreases following host attachment. Changes in these plant hormones regulate a variety of physiological activities, including the biosynthesis of secondary metabolites, metabolic pathways, plant hormone biosynthesis, hormone signal transduction, zeatin biosynthesis, tryptophan metabolism, circadian entrainment, and more (**Figure 5**). In root hemiparasitic plants like *M. oleifera*, the regulatory effects of plant hormones play a crucial role in plant development after the haustoria establish a connection with the host. Although our study did not measure the changes in hormone content in *M. oleifera* roots following parasitism, we can infer from the transcriptome data that the hormones in the roots will coordinate with those in the haustoria to regulate plant growth.

During the process of establishing a parasitic relationship between a hemiparasitic plant and its host, the spatiotemporal expression of genes, accumulation of proteins, changes in hormone levels, and the development of the haustorium are all regulated in a coordinated manner. In the process of seed germination and parasitism establishment by *Psittacanthus schiedeanus* on host branches, the expression levels of genes from different categories exhibit a regular and continuous upregulation or downregulation (Ibarra-Laclette et al., 2022). Similarly, during the infection of mesquite by *P. calyculatus*, the activity of cell wall-degrading enzymes shows stage-specific patterns: cellulase and β-1,4-glucosidase play a dominant role during haustorium development, while xylanase, endo-1,4-β-glucanase, and protease are significantly activated during the haustorium penetration and vascular connection stages; Plant hormones, such as auxins and cytokinins, exhibit spatial concentration gradients and are directly involved in the regulation of haustorium development (Aguilar-Venegas et al., 2023). This study primarily monitored the overall growth of the plant and did not specifically observe the stages of haustorium development. Consequently, the current transcriptomic sequencing and hormone content data reflect a composite of the entire haustorium development process, which may dilute the gene expression characteristics and hormone dynamic changes specific to certain developmental stages. However, the gene expression patterns and hormone regulatory networks that consistently promote plant growth should remain relatively stable throughout the monitoring period. The significant upregulation and downregulation of genes in the haustorium and roots of S1H-treated plants (**Supplementary Table S7**) reflect the overall changes occurring in the root system after parasitism.

### 4.3 Cultivation of *M. oleifera*

Due to the unique challenges of cultivating parasitic plants, they are rarely grown or introduced through artificial means (Thorogood et al., 2022). However, many parasitic plants have significant and unique economic value, and cultivating these plants for sustainable use remains a challenge in plant cultivation technology (Pignone and Hammer, 2016). It took 150 years of research into *Santalum album*, a representative root hemiparasitic tree, to uncover its host preferences, parasitic growth patterns, haustorial physiological characteristics, and host adaptations to parasitism (ArunKumar et al., 2022). Over more than 40 years of artificial cultivation, the habits of *M. oleifera* have gradually become understood, particularly after the discovery of its semi-parasitic root characteristics. However, despite its significant economic value, sophisticated and effective cultivation practices are still lacking.

To advance the development of *M. oleifera* cultivation techniques, we propose four key measures based on our research, our prior cultivation experience, and other studies on root semi-parasitic plants (1) After planting, *M. oleifera* should be able to quickly establishe a parasitic relationship with the host plant, ensuring that the seedlings acquire the necessary nutrients for early growth. Our study reveals a significant positive correlation between the growth of *M. oleifera* and the number of haustoria attached to the host roots. Other studies have shown that, as nutrient reserves in the roots of *M. oleifera* seedlings gradually deplete over the first growing season (Chen et al., 2024), the seedlings’ ability to establish parasitic relationships with other potential host plants also declines. When artificially selecting host plants, it is essential to consider not only the plant species (Matthies, 2017) but also its age (Moncalvillo and Matthies, 2023) and proximity (Thyroff et al., 2023) to *M. oleifera*. (2) The host plant should demonstrate strong environmental adaptability and have minimal growth requirements, ensuring its robust development and creating favorable conditions for the parasitism of *M. oleifera*. This is because the parasitic plant consumes a significant amount of the host plant’s water and nutrients. Strong adaptability of the host plant to adverse conditions ensures its normal growth while providing suitable conditions for the parasitic plant (Press et al., 1998; Zhang et al., 2023). In our cultivation of *M. oleifera*, we observed that during drought stress, the host plant wilts and eventually dies, subsequently threatening the survival of *M. oleifera*. (3) The host plant should grow rapidly, be vigorous, and be relatively larger than the parasitic plant, thereby providing sufficient nutrients to support the parasitism of *M. oleifera*. Our research shows that the aboveground biomass of *M. oleifera* is significantly and positively correlated (P<0.01) with that of *T. diversifolia*, and this relationship has been confirmed in other studies as well (Hautier et al., 2010; Matthies, 2017). (4) The host plant should be resilient to top pruning, which can promote the growth of *M. oleifera* by artificially adjusting the distribution of light and nutrients between it and the host plant. The rapid growth of host plants can enhance the growth of parasitic plants; however, excessive growth may block light and deplete nutrients. This contradiction can be managed by artificially pruning the tops of the host plants.

## 5 Conclusions

The efficiency of *M. oleifera* seedlings in directly absorbing nutrients from the soil is quite low, making it difficult for the plants to grow normally without a host. There is a significant positive correlation between the aboveground biomass of *M. oleifera* seedlings and the biomass of the host plant, indicating that, in order to effectively promote their own growth, *M. oleifera* seedlings must establish a parasitic relationship with a rapidly growing host larger than themselves. Once the seedlings establish a parasitic relationship with the appropriate host, the host plant promotes the growth rate of the aboveground parts of *M. oleifera* seedlings significantly more their belowground parts. This leads to an imbalance in growth between the aboveground and belowground parts of *M. oleifera*, which poses potential a risk to the survival of the hemiparasite if the host weakens or dies.

Transcriptome analysis revealed that parasitism induces significant changes in haustorial physiology, with up-regulated genes predominantly linked to hormone metabolism, stress responses, antibiotic biosynthesis, and lipid metabolism. These changes suggest an active metabolic response to support nutrient acquisition and defense. The hormone levels in haustoria parasitizing other host plant species undergo significant changes, influencing various metabolic processes in *M. oleifera* and ultimately impacting its growth. Moreover, the physiological activity of the roots undergoes significant changes following the establishment of the *M. oleifera*-host association, with a marked enhancement in the hemiparasite’s response to biotic and abiotic stresses, as well as improvements in basic metabolic processes. These changes suggest that host attachment strengthened the overall adaptability, nutrient synthesis, and stress resilience in *M. oleifera*, all of which are crucial for its growth, development, and survival. *M. oleifera* seedlings only transform from slow-growing plants into fully developed adults with normal physiological functions after establishing a parasitic relationship with a suitable host.

## Data Availability

The data presented in the study are deposited in the Genome Sequence Archive in National Genomics Data Center, China National Center for Bioinformation / Beijing Institute of Genomics, Chinese Academy of Sciences (accession number GSA: CRA027511) that are publicly accessible at https://ngdc.cncb.ac.cn/gsa.

## Supplementary material

Supplementary Data Sheet 1 Detailed experimental methods for RNA extraction and qRT-PCR.
