# Supplementary material for: Unveiling the mechanisms driving the rapid growth of Malania oleifera seedlings, a high-value root hemiparasitic plant
Source: Front Plant Sci. 2025 Jul 17;16:1589651. doi: 10.3389/fpls.2025.1589651 (PMC12310656; doi:10.3389/fpls.2025.1589651)
Supplement: Supplementary Data Sheet 1 — Detailed experimental methods for RNA extraction and qRT-PCR. [file DataSheet1.pdf]

1 RNA Extraction Method

(1) **Sample Preparation**:
Take approximately 100 mg of fresh plant tissue and place it in a 2 ml grinding tube. Grind the tissue using a grinder at 60 Hz for 60 seconds.

(2) **Addition of SL Buffer**:
After grinding, add 500 μl of SL buffer (ensure that β-mercaptoethanol has been added to the buffer before use). Immediately vortex vigorously to mix thoroughly.

(3) **Centrifugation and Column Loading**:
Centrifuge at 12,000 rpm for 2 minutes. Transfer the supernatant (350-400 μl) to a CS filtration column. Centrifuge at 12,000 rpm for 2 minutes again and collect the supernatant in a new RNase-free centrifuge tube.

(4) **Ethanol Addition and Column Binding**:
Add 0.4 times the volume of the supernatant in anhydrous ethanol and mix well (precipitation may occur). Transfer the resulting solution, including any precipitate, to the CR3 adsorption column. Centrifuge at 12,000 rpm for 15 seconds and discard the waste liquid.

(5) **Protein Removal**:
Add 350 μl of RW1 protein removal buffer to the column and centrifuge at 12,000 rpm for 30 seconds. Discard the waste liquid.

(6) **DNase I Treatment**:
Add 80 μl of DNase I working solution and incubate at room temperature for 15 minutes to digest any genomic DNA.

(7) **Protein Removal (Repeat)**:
Add another 350 μl of RW1 protein removal buffer and centrifuge at 12,000 rpm for 30 seconds. Discard the waste liquid.

(8) **Wash Step 1**:
Add 500 μl of RW wash buffer (ensure ethanol has been added before use). Centrifuge at 12,000 rpm for 15 seconds and discard the waste liquid.

(9) **Wash Step 2**:
Repeat step 8.

(10) **Drying the Column**:
Centrifuge the column at 12,000 rpm for 2 minutes with no added buffer to remove any residual wash solution. Add 30-50 μl of RNase-free water to the column and incubate at room temperature for 4-5 minutes to elute the RNA.

(11) **Final Centrifugation**:
Centrifuge at 12,000 rpm for 2 minutes. The RNA is now ready for immediate use or can be stored at -80°C for later use.

2 qRT-PCR Experimental Method

(1) RNA Extraction for qRT-PCR:
Total RNA for qRT-PCR was extracted using the same method as employed for

transcriptome analysis.

(2) Gene Detection with Primer and Probe Sequences:

The following primer pairs were used for gene amplification:

Primer Name	Primer Sequence	Position	Tm Value (°C)	Product Length (bp)
LOC131165241_1F	AATTGCTTCAGCTTTGTGGT	1953	63.6	147
LOC131165241_1R	TTGCCAGTGTTGATGTTAGC	2099	63.7	
LOC131144836_2F	AATGGTGGAGCTTGATTTCT	370	62.1	106
LOC131144836_2R	ATTAATGCCTCGGAAGCTAC	475	62.4	
LOC131166841_1F	CACTGGTGAGGAGCTTTGA	297	64.3	132
LOC131166841_1R	TACTGGTTGTGGCTGGAAG	428	64.4	
LOC131164606_2F	TTCTCCACACCATCAGGAA	439	63	139
LOC131164606_2R	TGAAGACATGATCACAACTGG	577	62.8	
LOC131146306_1F	AGGATACGAGTTGCTGAAGAA	571	63.7	111
LOC131146306_1R	TCCTCCAAACACCTGTCAA	681	63.6	
LOC131159712_3F	ATATGTGGCCGCTAGCTTAC	560	64.6	150
LOC131159712_3R	CTTTCCCTTTCCTGCTGAC	709	63.1	
LOC131166883_1F	ATCTGCTGCTTCAACTACCC	600	64.7	114
LOC131166883_1R	ACGTCTGAGGACAAACCTTC	713	64.6	
LOC131159619_3F	CTGATATCGGCGAAGGAGT	694	63.7	173
LOC131159619_3R	CAACCAACCTGGTGAGAAA	866	62.6	
LOC131158404_1F	AACCACCACCATTCATAACC	118	62.8	147
LOC131158404_1R	TGCAATGAACAAGACTGCTAA	264	63	
LOC131166337_3F	AGGGAAGAATGAGGACTGC	977	63.9	134
LOC131166337_3R	CTAATGGGAAGCGTCTCTGT	1110	64.1	
LOC131163893_1F	GCATCCTTATTTGCCAGAGT	2466	62.9	133
LOC131163893_1R	TCAACCCAGGGAGTCTTAAA	2598	63	
LOC131157378_2F	AGAGGTCCAACTTCTCCCA	308	64.7	78
LOC131157378_2R	GGCTCAGATCTCCAAAGGT	385	63.9	
LOC131162920_2F	GATCTTGAGCTCACTCTTGGA	1216	64	73
LOC131162920_2R	TATCCACCGCCAAGTACAG	1288	63.9	
LOC131161710_3F	GACGAGTTTGAGACCGATG	1120	62.9	106
LOC131161710_3R	CATAAGAAGGCCACTGTTTGT	1225	63.3	
LOC131147577_2F	GGAATGGGAGCTTACATGA	1710	61.6	116
LOC131147577_2R	TCTCAACACAACAAAGATTCG	1825	61.5	
LOC131168160_1F	GCTGTTGATCTGGTTGTGAA	1276	63.3	112
LOC131168160_1R	CTGGCCTGCTTATGCTATG	1387	63.1	
actb_1F	GCTATGTCAAGTGCGGTTT	423	63.4	117
actb_1R	TATCCTTCAGCTCCTGTTCC	539	63.5	

For this experiment, 1-2 pairs of primers were designed for each gene. qPCR (SYBR Green I) primer optimization results show that these primers have a single peak in the melting curve at 60°C, indicating no non-specific amplification, and they can be used in this experiment.

(3) **Reverse Transcription System and Program**

Preparation of buffer mixture:

- FQ-RT Primer Mix: 2 µl
- 10×King RT Buffer: 2 µl
- FastKing RT Enzyme Mix: 1 µl
- RNase-Free dd H_2O: 5 µl

Reverse Transcription System: 20 µl

- RNA: 1 µg
- Buffer mix: 10 µl
- H2O: Add to 20 µl

Reaction Program:

- 25°C for 5 minutes
- 42°C for 30 minutes
- 85°C for 5 minutes

(4) **SYBR Green I Setup**

For a 96-well plate (20 µl total reaction volume):

- Mix: 10 µl
- Primer_F: 0.5 µl
- Primer_R: 0.5 µl
- cDNA: 1 µl
- H2O: 8 µl

(5) **PCR Reaction Program**:

**Step**	**Temperature**	**Time**	**Cycles**
Pre-denaturation	95℃	4 min	1
Denaturation	95℃	10 sec	40
Annealing/Extension	60℃	40 sec	
Melt Curve	95℃	15 sec	1
	60℃	60 sec	
	95℃	30 sec	
	60℃	15 sec	
